# CNN with Pose Segmentation for Suspicious Object Detection in MMW Security Images

**DOI:** 10.3390/s20174974

**Published:** 2020-09-02

**Authors:** Zhichao Meng, Man Zhang, Hongxian Wang

**Affiliations:** 1 National Laboratory of Radar Signal Processing, Xidian University, Xi’an 710071, China; zchaoMeng@stu.xidian.edu.cn (Z.M.); hxwang@mail.xidian.edu.cn (H.W.); 2 The School of Physics and Electronic Engineering, Guangzhou University, Guangzhou 510006, China

**Keywords:** millimeter-wave image, security check, object detection, human pose segmentation, convolution neural network

## Abstract

Millimeter-wave (MMW) imaging scanners can see through clothing to form a three-dimensional holographic image of the human body and suspicious objects, providing a harmless alternative for non-contacting searches in security check. Suspicious object detection in MMW images is challenging, since most of them are small, reflection-weak, shape, and reflection-diverse. Conventional detectors with artificial neural networks, like convolution neural network (CNN), usually take the problem of finding suspicious objects as an object recognition task, yielding difficulties in developing large-amount and complete sample sets of objects. In this paper, a new algorithm is developed using the human pose segmentation followed by the deep CNN detection. The algorithm is emphasized to learn the similarity with humans’ body clutter applied to training corresponding CNNs after the image segmentation base of the pose estimation. Moreover, the suspicious object recognition in the MMW image is converted to a binary classification task. Instead of recognizing all sorts of suspicious objects, the CNN detector determines whether the body part images present the abnormal patterns containing suspicious objects. The proposed algorithm that is based on CNN with the pose segmentation has concise configuration, but optimal performance in the suspicious object detection. Extensive experiments confirm the effectiveness and superiority of the proposal.

## 1. Introduction

More security checks have been deployed to react the high-risk security environment due to the ongoing threat of terrorism [1]. Traditional security-check measures, such as X-ray equipment, arched metal detectors, and manual inspection, however, have shortcomings. For example, X-rays harm the human body, arched metal detectors only discern metal objects, and manual inspection poses the risk of personal discomfort. The millimeter-wave (MMW) three-dimensional imaging scanner [2,3,4,5,6,7] based on the near-field synthetic aperture radar (NF-SAR) three-dimensional imaging technology [8,9,10,11] offers an alternative. When compared with the traditional security-check measures, the MMW three-dimensional imaging scanner can provide the following advantages: 1. No X-ray radiation concern. The millimeter-wave frequency ranges from 3 GHz to 300 GHz, which is much lower than that of X-rays [12]. 2. Non-contact inspection. This MMW three-dimensional imaging scanner utilizes MMWs to inspect individuals passing through a line without subjecting them to physical contact. Different materials reflect MMWs in characteristic ways, and the captured waves can be processed to assess whether a suspicious object is present. 3. Clothing penetrability. Active MMW three-dimensional imaging scanner can see through clothing to imaging suspicious objects. Therefore, based on the application requirements of the MMW three-dimensional imaging scanner, studying the automatic object detection and recognition algorithms for the MMW image is of great significance.

## 2. Related Work

At present, there are two classes object detection methods for the MMW image: image threshold-based methods [13,14,15] and machine learning-based methods [16,17,18,19].

The image threshold-based methods mostly use the image gray histogram to determine the segmentation threshold, according to which the body and objects can be separated from each other, and then classify the objects. For example, both [20,21] proposed a two-level thresholding method for estimating the size of the concealed objects, where the lower threshold determines the regions of the human body and the higher one is used to segment the concealed objects. The advantage of these methods is the simple operation and low computational complexity. The literatures [22,23,24,25,26] use the Gaussian mixture model to model the image gray histogram and combine other algorithms (such as EM (expectation-maximization)) to calculate the segmentation threshold. These modeling-based methods are more reasonable and they can more accurately obtain a gray histogram. However, the performance of the modeling-based approaches is greatly affected by the mismatch of the model. Different from the above methods, the literatures [27] developed a new real-time algorithm that is based on the correlation function that characterizes the correlation between the standard properties of the suspicious objects and the properties of the MMW human images. The algorithm has a high detection accuracy when the template images are sufficient. Further, it can be seen that the performance of the algorithm is related to the number and quality of the templates. Moreover, this algorithm can be implemented unless the scattering intensity of the objects is stronger than that of the human body. Unfortunately, this premise cannot be always ensured, because the back-scattering intensity of the human bodies and suspicious objects may fluctuate greatly due to some factors, such as the different body parts, body fat content, the material of suspicious objects, the antenna illumination angle, etc. The fluctuation will highly influence the gray histogram of the final MMW images. Therefore, enhanced approaches are required in order to solve these problems.

In recent years, machine learning-based algorithms, including statistical machine learning [28,29,30] and deep neural networks [31,32,33,34,35,36,37], have been widely applied in the suspicious object detection for the MMW image. The literature [38] proposed a method that combines image processing with statistical machine learning techniques. This method had an anti-noise ability and it performed well on the poor-quality images. However, its multiple classifiers greatly increase the complexity of the algorithm. The object detection that is based on simple classifiers usually performs poorly in complex clutter scenarios, while deep neural networks potentially provide a powerful alternative. In [39], through a series of experiments, it is verified that the deep CNN (Convolution Neural Network) architecture is immune from the noise in the MMW image classification. Further, the multi-scale information of images is used to accurately classify. The detection method with a two-stage classifier in literature [40] was implemented in order to recognize the suspicious objects in THz images at a price of high computational complexity. Literature [41] proposed a high-performance detection algorithm that combines the complementary advantages of MMW images and visible images. The algorithm can generate high-precision human body profiles and accurately locate suspicious objects. However, combining the corresponding visible images increases the complexity of both the devices and the algorithm. Literature [42] proposed an algorithm that is applied to the dilated convolution to enlarge the spatial resolution of the feature maps. The algorithm works better for the small object detection, having a high detection rate and low false alarm rate in their data-sets. The paper [43] proposed a novel MMW image detection framework that is based on the well-known two-stage Faster-R-CNN pipeline. The algorithm achieved better performance on both precision and recall. In addition, the algorithms proposed in literature [44,45] are dedicated to locating and classifying the hidden objects in the MMW human images. Lei Pang et al. [46] introduced the YOLOv3 algorithm into concealed object detection, which is an one-step detection algorithm, and real time and high accuracy detection is realized. The algorithm, in fact, still obeys the same detection procedure as previous algorithms, i.e., positioning object, conferring contour, objects segmenting, and objects classification. Therefore, they have the same merits and drawbacks. Each of these algorithms based on the current CNN architecture usually has a standard and clear design framework. However, there are challenges lying ahead for these methods due to the following characteristics of suspicious objects. 1. Diverse shapes of the suspicious objects [47]. Due to the diverse shapes, we need enormous training samples to ensure category integrity. Moreover, the lack of color and texture information also creates the difficulty for the multi-category classification of MMW images. 2. Diversity of electromagnetic wave reflection of different suspicious objects [48]. Reflection intensity of the objects will be affected by their material, shapes, posture, and especially illumination angles. It is almost impossible to obtain the complete reflection information in training samples, which definitely weakens the performance of detecting the suspicious objects. 3. Strong and structure-complicated body clutter (the human body image rather than the object image) [49]. Suspicious objects do not necessarily reflect stronger than the body does due to the aspect sensitivity of the scattering reflection. Hence, strong body clutter may obscure small and reflection-weak objects. Moreover, due to their complex structure, some parts of the body significantly resemble suspicious objects, leading to detection errors.

For the problems in the MMW images to detect suspicious objects, an algorithm that is based on deep CNN detection integrated with human body pose segmentation is proposed in this paper. The proposed algorithm solves those challenges through the following improvements. 1. We convert the object recognition to binary classification task—anomaly detection, which allows for us ignore the diverse shapes of the suspicious objects because we no longer need to classify them. 2. The MMW imaging anomaly detection algorithm is more emphasized to learn the similarity with humans’ body clutter rather than the anomalous object, which avoid facing the second challenges directly and whose advantages as following. The second challenge lies in the large amount requirement on training data of diverse targets. However, large data sets with correct labels are deeming difficult to collect in MMW radar applications. On the contrary, data of body clutter are abundant and easy to collect. Furthermore, the characteristics of body clutter are easier to learn than that of objects. 3. The proposed algorithm reduces the risk of misjudgment through image segmentation and emphasizes the similarity with humans’ body clutter. In traditional methods, entire images are sent to the object detector. Subsequently, the detector may misjudge a human joint similar to objects as the object, which is unavoidable. However, in the proposed method, negative samples only contain body clutter that is background, if the detector misjudges a human joint as object there will be no background on this image, which is obviously a mistake. Therefore, as mentioned earlier, our detector emphasizes body clutter to avoid this mistake for segmented images.

In summary, our algorithm takes body clutter as the object of study and focuses on learning body clutter features and detecting clutter anomaly, which is the essence of the proposed algorithm. The main contributions of this paper are concluded as follows.

1. The clutter anomaly detection instead of suspicious object recognition paves a new way to the MMW imaging security check. In this way, on the one hand, sufficient training samples are readily accessible to us for learning body clutter features. On the other hand, the detector is robust to the shapes and reflection intensity of suspicious objects, because the proposed detector only aims at body clutter.

2. Great reduction of algorithm complexity. The complexity of the algorithm is greatly reduced, because the detector only performs the binary classification of body part images. The image segmentation and sufficient body clutter samples help to simplify the clutter anomaly detector as well as ensure satisfactory performance.

3. Stronger generalization capability is a potential contribution to the work. The clutter anomaly detector only aims at the body clutter. Thus, the detector will work effectively no matter what kind of suspicious objects are present. In other words, the detector has a stronger generalization capability.

The paper is organized, as follows: In Section 3, the Characteristics of MMW human images are analyzed, meanwhile the suspicious object detection algorithm that uses the human body pose segmentation followed by deep CNN detection is developed. In Section 4, experimental results with real measured data are given. Section 5 concludes the paper briefly.

## 3. The Proposed Algorithm

**Figure 1:**
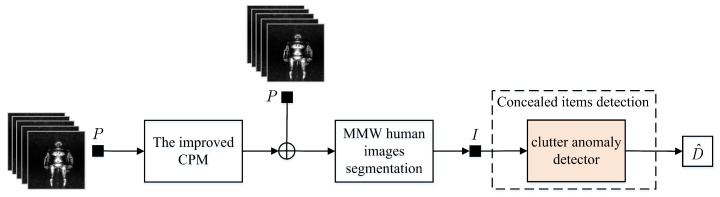
The proposed algorithm block diagram.

The complete body MMW image should be segmented into body part images for the individual body parts in order to remove the individual parts that are similar to objects in a MMW image and make the detector learn the features of body clutter more easily. Convolution pose machine (CPM) [50] is chosen in this paper to estimate human body posture. Based on the estimation, the coordinates of human joints in the MMW image can be obtained in order to segment complete human images into the body part images. Subsequently, we can discern the objects through detecting the clutter anomaly in every body part images. When compared with the conventional algorithms, the computational complexity of detection problem in this proposed algorithm has been reduced due to the image segmentation. Therefore, this detector is a lightweight neural network, thus improving the computation efficiency. Figure 1 and Algorithm 1 show the algorithm block diagram and flow separately.

**Algorithm 1** Convolution neural network (CNN) with Pose Segmentation.**Input:**
Complete MMW human images *P*.
**Start:**
 1:Initialize the improved CPM with the pre-trained weights and biases and initialize suspicious objects detection network with stochastic weights and biases. 2:Train the improved CPM until convergence and obtain the well-trained weights and biases $\left[W{}_{\mathrm{icpm}},B{}_{\mathrm{icpm}}\right]$. 3:Run the improved CPM with the well-trained weights and biases $\left[W{}_{\mathrm{icpm}},B{}_{\mathrm{icpm}}\right]$ and obtain coordinates of human joints: $\hat{J}=\psi \left(\left[W{}_{\mathrm{icpm}},B{}_{\mathrm{icpm}}\right],P\right)$. 4:Segment the complete MMW human images into body part images: $I=f{}_{\mathrm{seg}}\left(\hat{J},P\right)$. 5:Train the suspicious objects detection network until convergence and obtain the well-trained weights and biases $\left[W{}_{\mathrm{detector}},B{}_{\mathrm{detector}}\right]$. 6:Run the suspicious objects detection network with the well-trained weights and biases $\left[W{}_{\mathrm{detector}},B{}_{\mathrm{detector}}\right]$ on body part images: $\hat{D}=F{}_{\mathrm{detector}}\left(\left[W{}_{\mathrm{detector}},B{}_{\mathrm{detector}}\right],I\right)$.
**Output:**
Detection result: $\hat{D}$.


### 3.1. Human Posture Estimation and Image Segmentation

**Figure 2:**
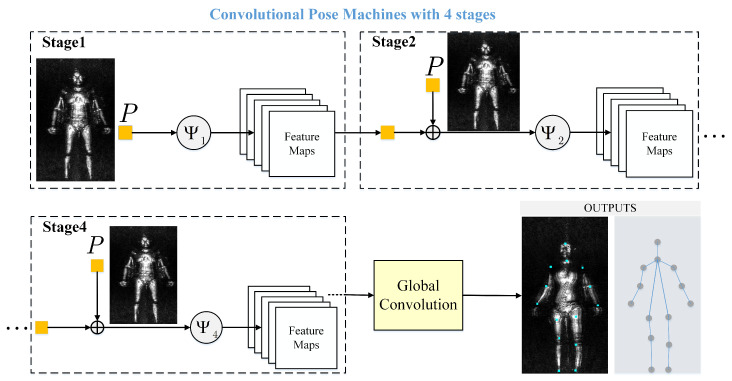
Improved convolution pose machine (CPM) with four stages. The original CPM is reduced from six stages to four stages and the global-convolution (GCN) layer is used rather than fully-connected (FCN) layer.

CPM was proposed by Shih-En Wei in 2016 [50]. CPM consists of a sequence of CNN predictors, such as stage 1 in Figure 2, is trained to make dense predictions at each image location. The convolutional network operates directly on the belief maps in the previous stage, and output increasingly refined joint point position estimation results. Because the original CPM has six stages, in order to prevent the gradient from disappearing, the authors use an intermediate supervision layer to ensure that in order to be able to generate increasingly accurate belief maps. However, there are some differences in our task. On the one hand, the original CPM with six stages has complexity structure and powerful fitting ability. The human poses in MMW human images, however, are simpler than that in visible images. Therefore, the original CPM is redundancy structure for our task. On the other hand, complexity structure and powerful fit ability make over-fitting more easily occurred on our small samples MMW image dataset than on the large number of visible image dataset. Therefore, it is necessary to simplify the original CPM to do the posture estimation.The structure and fitting ability are weakened; however, it is more suitable for our task. The structure of the simplified CPM is shown in Figure 2, and the details of Figure 2 are as follows.

1. Reduced four stages. In the MMW images, training samples are usually not sufficient for the original six-stage CPM. The original CPM needs to be reduced from six stages to four, which is more suitable for our task, when we apply it to estimating the human posture in order to avoid over-fitting.

2. The global-convolution (GCN) layer [51], whose scale of convolution is same as feature map, is used rather than the fully-connected (FCN) layer. Traditionally, the FCN layers are deployed at the end of networks. However, we choose the global-convolution layer since the GCN layer proves stronger expression ability and fewer weights than the FCN layer.

**Figure 3:**
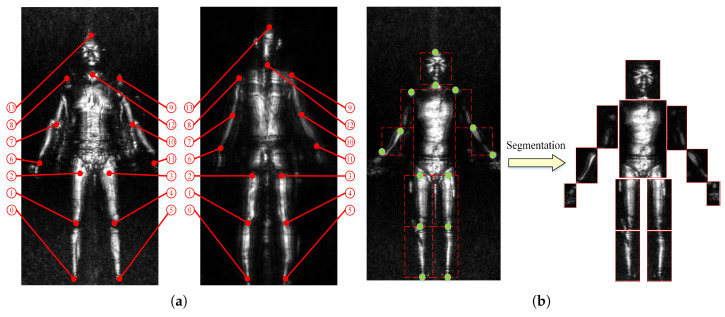
Human image marking and Segmentation rules. (**a**) Human joints marking rules. (**b**) Segmentation of human body image.

Training samples of human posture estimation include the images of the human body’s anterior and posterior surfaces. We mark the 14 joints of the human body such as the ankle, knee, waist, wrist, elbow, shoulder, neck, etc., and give a number to everyone, as shown in Figure 3a.

We segment the complete MMW human images after the posture estimation. Every complete MMW human image is divided into 12 body part images with the 14 joints, which is shown in Figure 3b. In practice, the images of the head, and palms are ignored because these parts cannot hide objects. Hence, the different body part images constitute the different sample sets separately. A lightweight network can be generated as the clutter anomaly detector on every body part image, owing to the segmentation.

### 3.2. Suspicious Object Detector

As mentioned earlier, more attention is shifted from suspicious objects to body clutter, which avoids the difficulties of learning the features of suspicious object. Correspondingly, clutter anomaly detection is chosen, rather than conventional suspicious object recognition, which makes good use of clutter information. For the clutter anomaly detection problem, an ensemble learning network detector that is based on two different lightweight networks is proposed. Utilizing the two networks not only avoids the redundancy of weights, but also benefits the detector through their combination. The final detection result comes from the decision fusion of the two networks’ outputs, improving the accuracy. In a security check, the leak-alarm, a false negative, proves to be more dangerous than the false-alarm. Therefore, in this paper, the logic “or” is used in the decision fusion in order to reduce the frequency of the leak-alarm, despite the fact that it may also increase the frequency of false-alarm.

**Figure 4:**
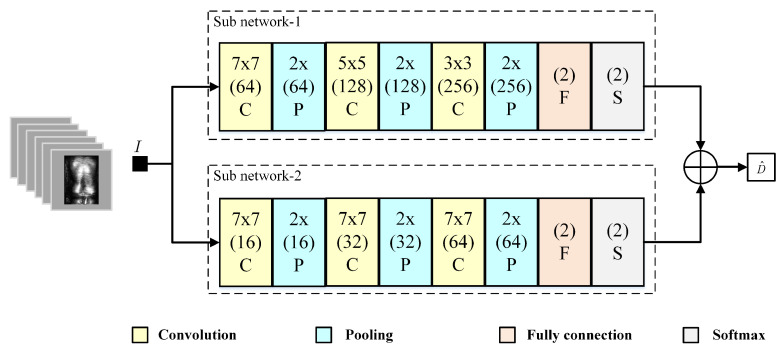
Structure of the clutter anomaly detector. Two different convolution structure are used for extracting the fine textures and the contour information of the body clutter respectively.

Figure 4 presents the structure of the clutter anomaly detector. The sub-network 1 is a four-layer convolution neural network, and the size of convolution kernels is reduced layer-by-layer, which is beneficial for extracting the fine textures of body clutter. The sub-network 2 has the same structure as the sub-network 1, while the convolution kernel size remains the same in its layers. The sub-network 2 mainly extracts the contour information of the body clutter, so that every convolution layer of the sub-network 2 has a large convolution kernel. In summary, the detection network can make good use of the contours and fine texture information of body clutter.

## 4. Experiments and Analysis

### 4.1. Experimental Dataset and Environment

**Figure 5:**
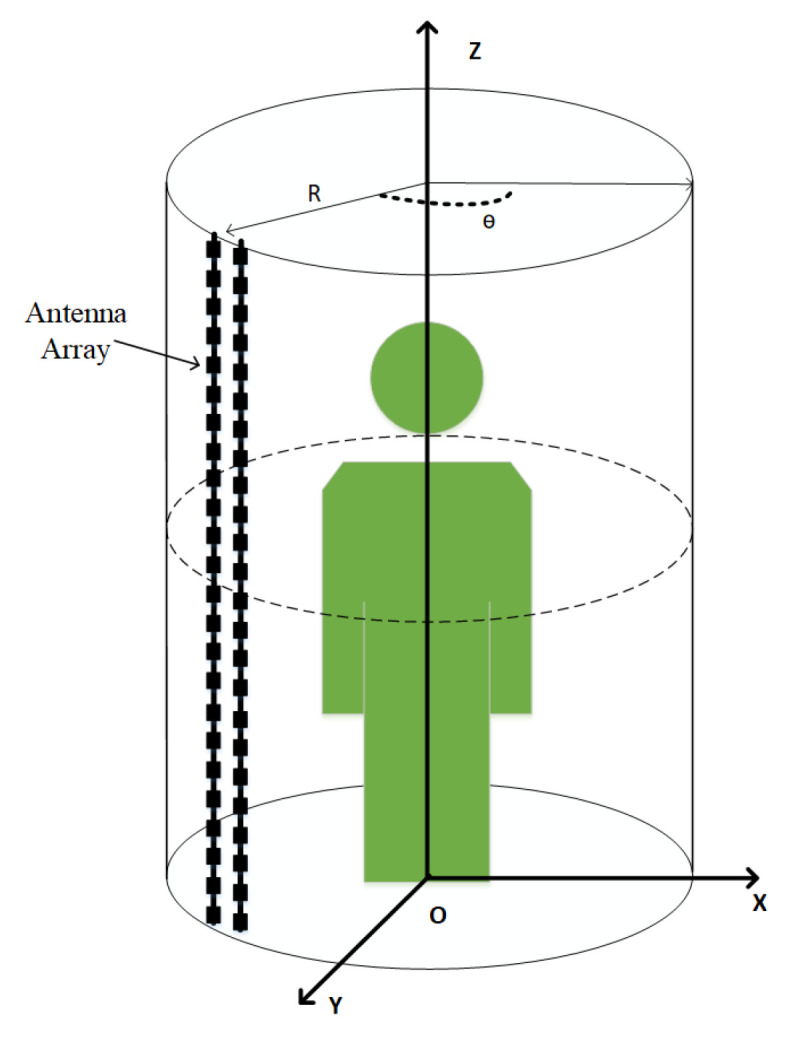
Model of experiment system.

An experimental radar system was built in order to obtain real MMW human images, and the model is shown in Figure 5. It works in ka band (27 GHz) with bandwidth 5 GHz. The experimental radar system’s range resolution, horizontal resolution, and vertical resolution are 20 mm, 5 mm, and 5 mm, respectively. Through the experimental radar system, approximately 3000 multi-angle MMW human images were obtained as the MMW human image dataset and each image takes the human body as a reference. Everyone has 12 different angles, including six angles on the body’s anterior and six angles on the posterior surfaces. There are four categories of objects in the dataset, including bottles, pistols, knives, and mobile phones, which are mainly located on the back, abdomen, waist, and legs of human bodies. Table 1 shows the number of objects in each category.

In all of the multi-angle MMW human images, the five hundred of them are firstly selected as the improved CPM training samples. Subsequently, the rest of them are segmented into body part images for every joint whose coordinates are obtained by the pose estimation resulting from the well-trained improved CPM. 1678 body part images are randomly selected as the samples for the training and testing of the detector from all of the body part images. In the 1678 body part images, the images containing suspicious objects were positive samples and the others were negative samples. Generally, the number of positive and negative samples should be roughly equal to that of negative ones [52,53]. Because suspicious objects are located on the back, abdomen, and legs, these samples consist of these three body part images. Table 2 shows the details about the samples.

All of the experiments were performed on a computer having a GTX 1080 GPU and 16 GB RAM to prove the effectiveness of the proposed method and compare the proposed algorithm with other algorithms.

### 4.2. Experiments and Discussion

**Figure 6:**
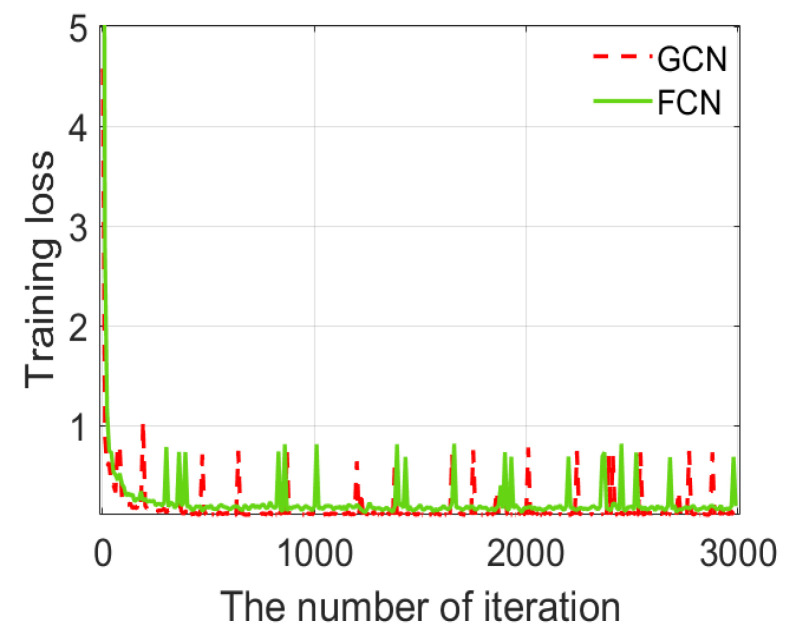
Training loss in the training process.

As a result of few training samples, a pre-trained CPM model on visible image datasets was used in order to initialize the improved CPM, which is called transfer learning. Based on the good initialization, the network’s weights also need to be tuned for the pose estimation on MMW human images. We use GCN layer rather than FCN layer in the improved CPM, whose size of convolution kernel is same as that of feature maps. In order to demonstrate the effectiveness of the GCN layer, we compare its converge performance with that of the FCN layer, which is usually applied in CNNs. Figure 6 shows the converge performance of the improved CPM based on the GCN layer and the FCN layer. The loss curve presents the fitting performance of the network. Faster convergence can significantly reduce the iterations of training. A smaller loss value indicates that the network can reach a better convergence state. From Figure 6, due to transfer learning is used in the improved CPM, loss curves begin with a small value. Although there are ups and downs during decline, both of the loss curves fall fast. The GCN layer has the faster converge speed and smaller loss value. Therefore, we can conclude that the improved CPM based on the GCN layer can converge the training loss function better and faster to ensure the pose estimation.

**Figure 7:**
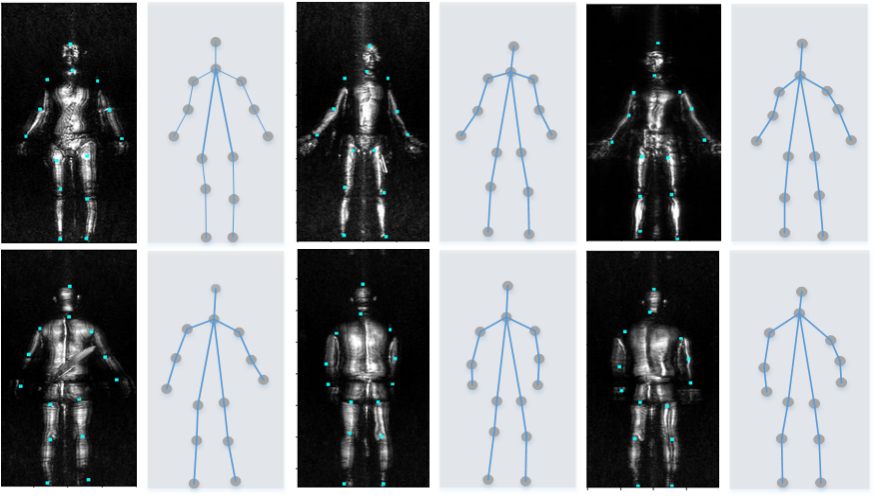
Results of the posture estimation.

However in practice, due to the fluctuation of the body reflection, there are always some human joints missing on MMW human images. To solve this problem, we expect the improved CPM to enable the accurate prediction of the coordinates of the joints missing on MMW human images, providing a prerequisite for the segmentation. The improved CPM network can correctly estimate every joint, as shown in Figure 7. The improved CPM can still predict their coordinates, even if some human joints do not appear in the MMW human images. After the improved CPM returns the joint coordinates for each MMW human image, the image segmentation is performed.

**Figure 8:**
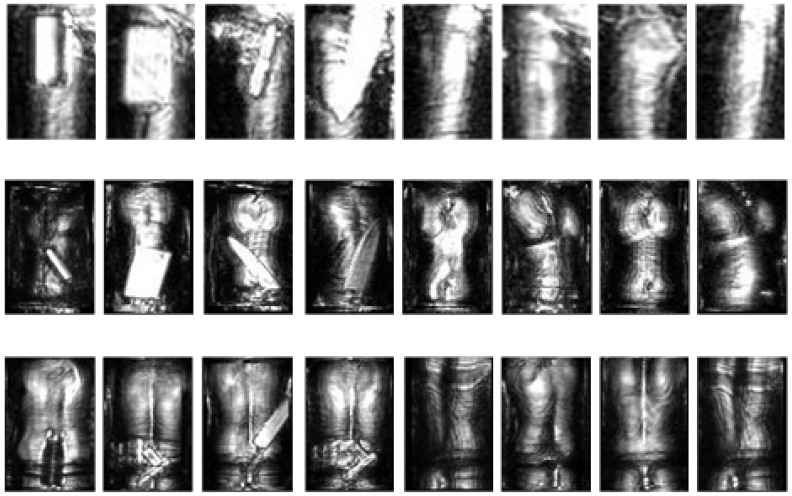
Body part images.

We segment the MMW human image into the body part images for the human joints. After this segmentation, the body part images are obtained, as shown in Figure 8. When compared with the complete MMW human images, the body clutter in the body part images becomes more monotonous and simple-structure, which is beneficial to learn its features. Besides, in the image of an individual body part, suspicious objects are more easily distinguished from the human body, thus reducing the frequency of false-alarm. In our MMW human images, the suspicious objects are located on the thigh, abdomen, and back of the body, whose images are displayed in Figure 8. Correspondingly, our algorithm is verified through these body part images.

**Figure 9:**
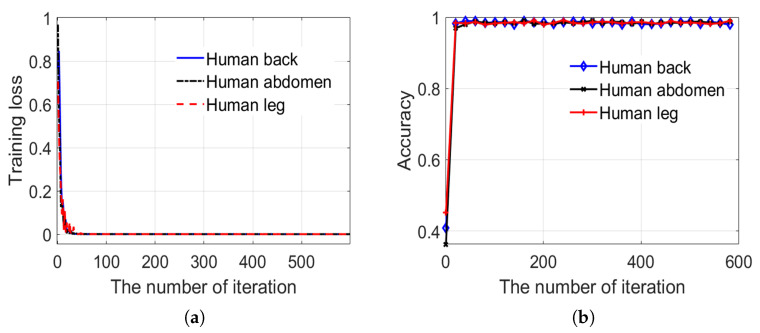
Variation of training loss and testing accuracy in the training process of sub-network 1. (**a**) training loss in the training process. (**b**) testing accuracy in the training process.

**Figure 10:**
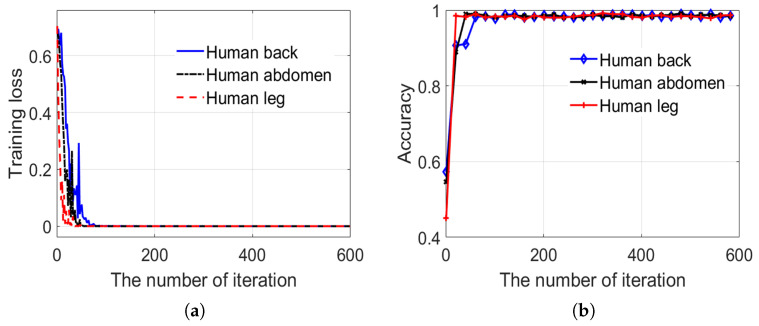
Variation of training loss and testing accuracy in the training process of sub-network 2. (**a**) training loss in the training process. (**b**) testing accuracy in the training process.

Clutter anomaly detection aims at every single body part image. The 1678 body part images, which include the thigh, abdomen, and back of the body have been obtained as a dataset, as mentioned earlier. The dataset is divided into two parts, namely a training set and a test set, which account for 82% and 18%, respectively. Our detector training and testing results are shown in Figure 9 and Figure 10. Figure 9 shows the results of the detector training and Figure 10 shows the results of the detector testing.

Through average 40 iterations, the loss function of sub-network 1 in the detector is converged to the minimum point, and the graph of loss function dithers near the minimum point, as shown in Figure 9a. Subsequently, the training is not completed until 600 iterations in order to ensure convergence stability. It can be seen that no sooner has the iteration started than the graph reaches the minimum point, which demonstrates the high convergence speed, meanwhile Figure 9b shows that the test accuracy of the network varies with the number of iterations. With the network converging, the test accuracy also increases rapidly. Finally, after the network has been converged, the accuracy rate remains at about 98%.

Figure 10 shows the training and testing results of the sub-network 2 in the detector. Like sub-network 1, the sub-network 2 is converged to a minimum after iterations and the accuracy also reaches a maximum. However, the fluctuation of the loss function during the training of sub-network 2 was severe, which means that using detail textures to detect suspicious objects is easier than using contour texture information. The two sub-networks are combined for the decision fusion in order to achieve higher detection rate and lower the false alarm probability; the simple and effective logic “or” was chosen as the fusion mechanism. Ultimately, the accuracy of the detector on the testing set is 98.5%.

**Figure 11:**
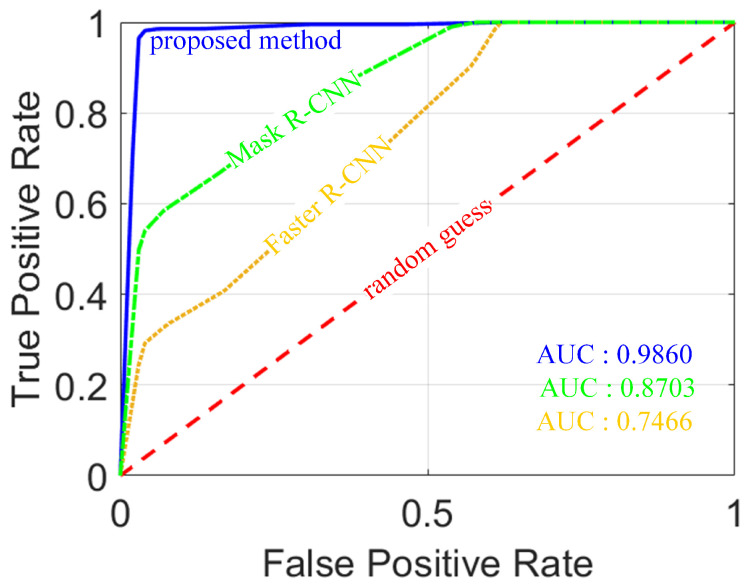
Receiver Operating Characteristic (ROC) curves of three methods.

The proposed algorithm was compared with Faster R-CNN and Mask R-CNN to further demonstrate the advantages of the proposed algorithm. During the training process, each algorithm is guaranteed to reach a convergence state. The performance metrics of each algorithm are shown in Table 3 and Table 4, and Figure 11.

The strategies are evaluated with the confusion matrix. Table 4 lists the important evaluation indicators.

The ACC (Accuracy) is defined as

(1) $$\mathrm{ACC}=\frac{\left(\mathrm{TP}+\mathrm{TN}\right)}{\left(\mathrm{TP}+\mathrm{TN}+\mathrm{FP}+\mathrm{FN}\right)}$$

It reflects “the ability of the classifier to determine the entire sample correctly”. The precision is the proportion of “the number of correct prediction data in those data predicted to be positive”. The recall is the percentage of “the number of correct predictions in positive samples”. PPV (Positive Predictive Value), TPR (True Positive Rate), FPR (False Positive Rate), F1 (balanced F Score, which is defined as the harmonic average of precision rate and recall rate) and MCC (Matthews Correlation coefficient) are defined as

(2) $$\mathrm{PPV}=\frac{\mathrm{TP}}{\mathrm{TP}+\mathrm{FP}},\mathrm{TPR}=\frac{\mathrm{TP}}{\mathrm{TP}+\mathrm{FN}}$$

(3) $$\mathrm{FPR}=\frac{\mathrm{FP}}{\mathrm{FP}+\mathrm{TN}},F1=\frac{2\mathrm{TP}}{2\mathrm{TP}+\mathrm{FP}+\mathrm{FN}}$$

(4) $$\mathrm{MMC}=\frac{\mathrm{TP}*\mathrm{TN}+\mathrm{FP}*\mathrm{FN}}{\sqrt{\left(\mathrm{TP}+\mathrm{FP})*(\mathrm{TP}+\mathrm{FN})*(\mathrm{TN}+\mathrm{FP})*(\mathrm{TP}+\mathrm{FN}\right)}}$$

where TP, FP, FN, and TN are the probability of true positive, false positive, false negative, and true negative respectively.

The recall of Mask R-CNN is 99.09%, which means that Mask R-CNN is better at learning the features of suspicious objects and detecting them, as shown in Table 4. Its PPV of 68.5% indicates that Mask R-CNN misidentifies body clutter as suspicious objects more easily. From its ACC of 74.83%, the performance of Mask R-CNN is not as good as we expected, meanwhile the performance of Faster R-CNN is inferior to Mask R-CNN. The low TPR and PPV indicate that its ability to extract effective features of suspicious objects and body clutter should be further improved. As we said, these algorithms pay more attention to suspicious objects rather than body clutter. Because suspicious objects have diverse shapes and diverse reflections, it is difficult to extract effective features of them. Besides, strong and structure-complicated body clutter cannot be ignored. Therefore, the errors in these algorithms seem inevitable.

On the contrary, the proposed algorithm is completely different from them, which pays more attention to body clutter rather than suspicious objects. By image segmentation, body clutter learning, and clutter anomaly detection, suspicious objects can be distinguished with higher accuracy. Firstly, the image segmentation not only removes the images of body parts which look like suspicious objects, but also makes the detector learn the body’s common features easily. Subsequently, clutter learning and clutter anomaly detection reduce the complexity of the suspicious item detection problem and enhance the generalization ability of the detector while making good use of body clutter information. Finally, our algorithm achieved the ACC of 98.5%, the PPV of 98.6%, and the TPR of 98.5%. These experiment results indicate the effectiveness of our algorithm. The F1 score and MMC also confirm the excellent performance of the proposed algorithm.

ROC (Receiver Operating Characteristic) indicates the performance, which curves with FPR as the abscissa and TPR as the ordinate. The area under curve (AUC) is equivalent to the probability that a randomly chosen positive example is ranked higher than a randomly chosen negative example. A larger value indicates better classifier performance. The ROC curve of the proposed algorithm is closer to the upper left corner, which means that the proposed algorithm has stronger classification ability, as can be seen from Figure 11. The AUC of this curve is 0.986, which is the largest among the three algorithms, so the classification performance of the proposed algorithm is better than the other two algorithms.

## 5. Conclusions

In MMW imaging, conventional detection methods based on learning features of suspicious objects face some difficulties including the strong and complex body clutter, the diverse shapes and the fluctuation electromagnetic reflections of objects. Clutter feature learning rather than suspicious object feature learning is applied in our algorithm, thus a novel suspicious object detection algorithm based on MMW human image segmentation followed by deep CNN detection is developed in this paper in order to overcome these difficulties. Firstly, the improved CPM is used for the pose estimation on complete MMW human images to obtain the coordinates of every joints. Subsequently, the complete MMW human images are segmented into the body part images. In these body part images, the body clutter becomes more monotonous and simpler in structure, which is beneficial for the subsequent detector. Finally, the lightweight CNN as a clutter anomaly detector is used to detect the suspicious objects on every body part image improving the detection effectiveness. We compare our algorithm with other popular algorithms in several aspects. It can be seen from the experimental results that the GCN layer are more effective than the FCN layer due to its better convergence performance. The improved CPM based on the GCN layer enables the accuracy prediction of the missing joints on MMW human images, as we expected. Through the contrast experiments, it is verified that our algorithm is more effective and our clutter anomaly detector has stronger generalization ability.

In the following works, we will try to use multi-look MMW images observed from one person to improve the detection ability in more complex environments.

## Figures and Tables

**Table 1:** Millimeter-wave radar human dataset.

Category	Total	Phone	Bottle	Pistol	Knife
**Number**	2440	84	480	960	916

**Table 2:** Suspicious objects Detection dataset.

Category	Back	Abdomen	Leg
**Negative Samples**	258	246	263
**Positive Samples**	339	268	300

**Table 3:** Confusion matrices of three methods.

Proposed Method		Faster R-CNN		Mask R-CNN	
894 (TP)	13 (FP)	605 (TP)	293 (FP)	898 (TP)	413 (FP)
754 (TN)	13 (FN)	474 (TN)	302 (FN)	354 (TN)	9 (FN)

**Table 4:** Detection performance with different algorithms.

Methods	ACC	PPV	TPR	FPR	F1	MCC
**Mask R-CNN**	74.79	68.50	99.00	53.85	0.81	54.60
**Faster R-CNN**	64.46	67.34	66.70	38.20	0.67	28.48
**Proposed Method**	98.45	98.57	98.57	1.69	0.98	96.87
